# Does a brief workshop change clinical associate students’ resilience?

**DOI:** 10.4102/phcfm.v8i1.1183

**Published:** 2016-09-15

**Authors:** David Rogers

**Affiliations:** 1 Department of Family Medicine, University of Pretoria, South Africa; 2 Department of Clinical Education, Plymouth University Peninsula Schools of Medicine and Dentistry, United Kingdom

## Abstract

**Background:**

Clinical associates resilience is important as many will work in adverse circumstances. There is some evidence that educational interventions can improve health care student resilience although it is conflicting. There is no previously published research on educational interventions for resilience in clinical associate students.

**Objective:**

To investigate whether a brief resilience workshop could improve resilience in clinical associate students.

**Methods:**

A single cohort pre-post design was used. Resilience scores were calculated using the Connor-Davidson 25-item resilience scale in a cohort of clinical associate students before and 8 weeks after a brief resilience workshop.

**Results:**

Although no statistically significant changes were observed after a brief resilience workshop, this study adds to the existing body of knowledge on resilience in African health care training.

**Conclusion:**

The evidence for education interventions to improve resilience is conflicting and complex. Given the relevance to health care workers and their educators, interventions to improve resilience should continue to be evaluated and the outcomes should be reported.

## Introduction

The term ‘resilience’ can be defined as the ability to recover from difficult situations, as well as the capacity to endure ongoing hardship.^1^ The benefits of enhanced resilience in health care workers include better clinical outcomes for patients, a reduction in the loss of medical personnel and improved personal well-being for the individual professional.^2^

The Bachelor of Clinical Medical Practice (BCMP) programme trains mid-level health care workers (clinical associates) to work in district hospitals and primary health care clinics. Initial reports on the output of the BCMP programme are that the graduates make a difference where they work.^3^ In spite of this, a substantial number of graduates report high levels of adversity.^4^ Sources of this adversity include the understaffed and poorly resourced clinical environments they work in, long delays in finding employment, lack of a clear scope of practice and challenges in being accepted into established health care teams.^5^

While solutions are being developed, improving student resilience could help graduates cope with adversity. Previous studies have suggested that education can enhance individual resilience through interventions, such as resilience workshops,^6,7,8,9^ cognitive behavioural training,^10,11^ mindfulness training^12,13^ and mentoring.^8^

There are currently no studies looking at resilience interventions in African medical education, and very little is known about resilience levels in health care students or professionals in an African context. This study aims to establish the resilience levels of final year BCMP students, and tries to ascertain whether brief workshop-based resilience training could change student resilience.

## Methods

### Study design

A single cohort pre-post design was chosen to establish BCMP student resilience scores, and whether these scores changed following a resilience workshop. A single cohort study can measure if any changes have occurred in students’ resilience although this study design is not able to answer whether the intervention led to any of the changes observed. Although more complex designs would have been ideal, they were not feasible.

### Study population

The study population consisted of volunteers from the 2015 final year BCMP students at the University of Pretoria (*n* = 62). With a continuous outcome variable such as resilience scores, a sample size of 40 or more provides statistical power (two-tailed, *α* = 0.05) of > 85% to detect a difference of one standard deviation between groups. As the final year total cohort was 69, the participation of 90% of students in this study suggests that the findings should be relevant to the whole cohort.

Baseline resilience scores were also collected from volunteer first-year (*n* = 36) and second-year (*n* = 48) students and other health care staff at the clinical learning centres (CLCs) (*n* = 68). This was done to collect background data on the resilience milieu within which BCMP students are trained.

### Intervention

A resilience workshop was developed from a review of the literature and with the help of a focus group at the 2014 Rural Doctors of South Africa (RUDASA) conference. A pilot study was conducted on a small group of BCMP students (2014 cohort). The feedback from the group was used to further improve the final workshop design.

The title of the workshop was ‘Educational Interventions to Enhance Personal Resilience’. The workshop was a 90-min session using a mixture of didactic teaching, a multimedia presentation, small group sharing and problem-solving, and reflection. The workshop covered the importance of resilience for health care professionals and stressed that developing resilience involved a commitment to self-care. The workshop utilised the following techniques aimed at improving resilience: reflection, small group problem-solving and sharing, teaching on cognitive behavioural and mindfulness techniques, and encouragement to form peer-mentoring networks. Each of these techniques was explained and discussed in some detail, and then the students were divided into smaller groups to decide how they were going to work on their own resilience in different CLCs during the coming year. Once each group had prepared an action plan, the plans were shared with the whole cohort.

The choice to use an action learning and problem-solving approach reflected a desire to motivate the students to own the responsibility for self-care and resilience. A similar workshop was also held for facilitators to ensure that they could support the students in this process.

### Study data

Resilience scores were calculated using the Connor-Davidson 25-item resilience scale (CD-RISC). This measure was chosen as it was rated the highest in a recent resilience scale review.^14^ While scoring highest in this review, the validity of the CD-RISC has been questioned in a study probing the development of resilience in a rural South African setting, which found that ‘a priori, Eurocentric conceptualizations of resilience are potentially flawed, particularly when seeking to understand how Black youth resile’.^15^

Despite these concerns, the CD-RISC has been used as a serial measure of resilience in many previous trials,^7,10,11^ the authors have shown good test–retest reliability (*r* = 0.87)^16^ and the CD-RISC has been validated in a rural South African context.^17^

The resilience scores of the BCMP students were assessed before the workshop using a paper version and again after 8 weeks using an email invitation to a confidential online version. While longer follow-up intervals could allow the detection of a late increase in resilience, they are associated with higher dropout rates, and an 8-week interval enables a comparison with previous resilience trials.^6,7,10,11^

### Quantitative data analysis

The mean CD-RISC scores before and after the interventions were compared using the two-tailed paired student’s *t*-test. This establishes whether a statistically significant change in the two means was observed, and is frequently used in medical research where the data have a normal distribution.^18^ An alpha level of 0.05 was used in keeping with the convention in medical education trials. Cohen’s D method allows for a calculation of effect size based on the data obtained from the paired student’s *t*-test, and was used to assess the effect size of the intervention.

### Ethical considerations

The study was approved by the University of Pretoria Health Faculty Research Ethics Committee (BCMP 56/2011).

## Results

The study population consisted of 62 final year BCMP students.

The age range was 18–28 (mean = 22). There were 35 females (56%) and 27 males (44%).

Of the total students, 43 completed the study, 18 did not respond to the follow-up survey invitation and 1 requested to opt out of the study. No reasons were given.

The participants who dropped out (10 females and 9 males, mean age = 22) did not differ significantly in their baseline responses to the study population (mean CD-RISC non-completing participants 80.21 ± 3.58 vs. study population 77.37 ± 2.14, *p* = 0.20). Table 1 summarises the data for the study.

**TABLE 1:** Summary of before and after scores.

Before and after	*N*	Mean CD-RISC scores	95% CI
Before	62	77.37	75.53–79.81
After	43	74.12	70.79–77.45
*P*-value	-	0.38 (> 0.05)	-
D-score	-	−0.33	-

*Source:* Authors’ own work

CD-RISC, Connor–Davidson resilience scale; CI, confidence interval.

## Discussion

BCMP resilience scores did not change significantly after the brief workshop. When compared to results from similar studies (Table 2), it is notable that while workshops were part of all the trials (except Sood and Sharma), other studies used multiple workshops over a longer time period (48 h to 4 weeks), and the total workshop attendance time was much higher (8–16 h vs. 1.5 h).

**TABLE 2:** Studies assessing resilience interventions.

Author	Intervention	*N*	Initial mean CD-RISC	Follow-up CD-RISC	*p*	Effect size (d)
Rogers	Brief workshop	62	77.4	74.1	0.38	−0.33
Peng^7^						
High resilience group	Pennsylvania	15	87.0	87.1	0.57	†
Low resilience group	Resilience Program	15	58.5	69.4	0.01	†
Pidgeon^13^	Mindfulness training	16	78.5‡	82.9‡	0.008	†
Fortney^12^	Mindfulness training	30	79.9‡	83.2‡	(> 0.1) †	†
Sharma^10^	SMART program	38	73.4	81.8	< 0.001	†
Sood^11^	SMART program	32	69.6	79.4	0.003	+1.16
Dolbier^19^	Resilience program	30	67.7	75.3	< 0.01	†

*Source:* Authors’ own work

CD-RISC, Connor–Davidson resilience scale. When studies reported on more than one cohort, only the data from the intervention cohort(s) are included.

† Not reported.

‡ 14-item resilience scale is used; therefore, CD-RISC scores are not directly comparable.

The brief nature of the BCMP workshop may have contributed to why no significant change was observed, although the Fortney trial had multiple long workshops, and also failed to report significant improvements in resilience scores.

Confounding effects in this study were possible because of its design and may have contributed to the non-significant outcome. For example, the intervention was done at the start of the final year, students then moved to a new clinical environment prior to the follow-up measurement and this could have resulted in a change in their scores.

The relatively high baseline score of the BCMP students may have been a factor. In a Chinese study^7^, the high initial resilience group also did not show any significant changes after the resilience programme.

A comparison of the baseline CD-RISC in this study and studies on other international health care students suggests that BCMP students show high baseline resilience (Table 3).

**TABLE 3:** CD-RISC scores in health care students.

Author	*N*	CD-RISC (s.d.)	Location	Cohort description
Steinhardt^6^	27	70.6 (12.3)	USA	University students
Peng^7^	1198	61.7(10.6)	China	Medical students
Stephens^20^	70	74.5 (†)	USA	Nursing students
Rogers	62	77.37 (8.6)	RSA	Third year BCMP students

*Source:* Authors’ own work

BCMP, bachelor of clinical medical practice; CD-RISC (s.d.), Connor–Davidson resilience scale (standard deviation); RSA, Republic of South Africa; USA, United States of America.

† Unreported data.

The baseline CD-RISC scores for the study population compared with other reported South African groups are shown in Table 4. The baseline scores for BCMP students in this study are within the range of scores reported in previous South African studies.

**TABLE 4:** CD-RISC scores in South African population.

Author	*N*	Mean (s.d.)	Location	Population description
Spies^21^	95	80 (†)	RSA	Women with HIV
Rogers	62	77.37 (2.14)	RSA	BCMP baseline scores
Hemmings^22^	150	73 (†)	RSA	Non-TB contacts of family members with TB
Fjeldheim^23^	102	65 (†)	RSA	Paramedic trainees without PTSD
Jorgensen^17^	701	64.8 (18.9)	RSA	Random adolescent sample

*Source:* Authors’ own work

CD-RISC, Connor–Davidson resilience scale; PTSD, posttraumatic stress disorder; RSA, Republic of South Africa; TB, tuberculosis; s.d., standard deviation.

† Not reported.

Table 3 and Table 4 suggests that BCMP students show a good baseline resilience in comparison to other international health care students, and their scores appear to be similar to that of other reported South African groups.

BCMP student resilience scores appear to be higher than that of some groups of heath care workers in the CLCs (Table 5). Future studies could compare resilience scores between these groups and explore how resilience changes over time.

**TABLE 5:** Resilience scores of clinical learning centre staff.

Group	*N*	CD-RISC (s.d.)
Doctors	18	68.7 (11.3)
Nurses	40	74.1 (15.4)
Allied	10	81.7 (8.6)
Third-year students	62	77.37 (8.6)
Second-year students	43	78.1 (10.7)
First-year students	36	73 (13)

*Source:* Authors’ own work

CD-RISC (s.d.), Connor–Davidson resilience scale (standard deviation).

Observational studies report on changes, but can only speculate on causes. No net resilience changes were observed in this cohort. This is likely to be the result of the positive and negative resilience factors being in balance over the time period. We could speculate that the final year programme is stressful and the resilience scores may have dropped if it were not for this brief workshop; however, this study does not provide any evidence to suggest that brief workshops are associated with significant changes to BCMP student resilience.

## Conclusions

Improving BCMP student resilience is important given the adverse working conditions that many graduates face. The literature suggests that education is a potential route to enhancing resilience. The evidence for education interventions to improve resilience is conflicting and complex. This study shows that relevant interventions must be evaluated and monitored and the outcomes must be reported. Although no statistically significant changes were observed after a brief resilience workshop, this study adds to the existing body of knowledge on resilience in an African health care training context.
